# Bio-Inspired Synthesis of Injectable, Self-Healing PAA-Zn-Silk Fibroin-MXene Hydrogel for Multifunctional Wearable Capacitive Strain Sensor

**DOI:** 10.3390/gels11050377

**Published:** 2025-05-21

**Authors:** Rongjie Wang, Boming Jin, Jiaxin Li, Jing Li, Jingjing Xie, Pengchao Zhang, Zhengyi Fu

**Affiliations:** 1 State Key Laboratory of Advanced Technology for Materials Synthesis and Processing, Wuhan University of Technology, Wuhan 430070, China; rjwang@whut.edu.cn (R.W.); 335933@whut.edu.cn (B.J.); 330987@whut.edu.cn (J.L.); finiteli@outlook.com (J.L.); zyfu@whut.edu.cn (Z.F.); 2 Hubei Longzhong Laboratory, Xiangyang 441022, China; 3 International School of Materials Science and Engineering, Wuhan University of Technology, Wuhan 430070, China

**Keywords:** polyacrylic acid, silk fibroin, Ti_3_C_2_T_x_ MXene, flexible, strain sensor, hydrogel

## Abstract

Conductive hydrogels have important application prospects in the field of wearable sensing, which can identify various biological signals for human motion monitoring. However, the preparation of flexible conductive hydrogels with high sensitivity and stability to achieve reliable signal recording remains a challenge. Herein, we prepared a conductive hydrogel by introducing conductive Ti_3_C_2_T_x_ MXene nanosheets into a dual network structure formed by Zn^2+^ crosslinked polyacrylic acid and silk fibroin for use as a wearable capacitive strain sensor. The prepared injectable hydrogel has a uniform porous structure and good flexibility, and the elongation at break can reach 1750%. A large number of ionic coordination bonds and hydrogen bond interactions make the hydrogel exhibit good structural stability and a fast self-healing property (30 s). In addition, the introduction of Ti_3_C_2_T_x_ MXene as a conductive medium in hydrogel improves the conductivity. Due to the high conductivity of 0.16 S/m, the capacitive strain sensor assembled from this hydrogel presents a high gauge factor of 1.78 over a wide strain range of 0–200%, a fast response time of 0.2 s, and good cycling stability. As a wearable sensor, the hydrogel can accurately monitor the activities of different joints in real-time. This work is expected to provide a new approach for wearable hydrogel electronic devices.

## 1. Introduction

Wearable flexible sensors have become a research hotspot in recent years due to their crucial applications in healthcare and human–machine interaction [1,2,3,4]. Conductive hydrogels, as a type of flexible three-dimensional network composed of polymer chains in a water-rich environment, can withstand mechanical deformation and other characteristics, and have increasingly been widely used in wearable flexible strain sensors for monitoring physical signals in human joint activities [5,6,7]. Capacitive strain sensors constructed of conductive hydrogel can efficiently and accurately convert biological signals into different states of electrical signals, thus meeting the important needs of wearable sensors [8,9,10]. For example, Gu et al. recently prepared a conductive hydrogel for flexible capacitive sensors by a physical kneading strategy, which has a sensitivity of 0.91 at a working strain of 190% [11]. Among them, the preparation of flexible hydrogels by ionic crosslinking usually has high flexibility, which has been widely studied.

Polyacrylic acid (PAA), as a typical water-soluble high polymer, is widely used in the preparation of hydrogel [12,13,14,15]. The large amount of carboxyl groups of polyacrylic acid can crosslink into a network structure through ion coordination, such as Ca^2+^, Zn^2+^, Ni^2+^, and Mg^2+^ [16,17]. For example, Ang Li et al. prepared a PAA hydrogel with high adhesion through Ca^2+^ crosslinking [18]. The ionic crosslinking method based on PAA provides a way to develop injectable self-healing hydrogels. In recent years, introducing a second network structure can effectively solve the weak ion crosslinking of single PAA hydrogel and construct a stable network structure [19,20]. Among them, natural biopolymers such as sodium alginate, chitosan, or hyaluronic acid were used to construct hydrogel flexible networks [21,22]. These materials have received considerable attention due to their intrinsic biocompatibility, biodegradability, and suitability for skin-interfacing electronics. For instance, Li et al. utilized sodium alginate to enhance polyacrylamide/xanthan gum double network ionic hydrogel for stress sensing and self-powered wearable device applications [23]. Silk fibroin, as a natural fiber biopolymer, possesses good flexibility and unique mechanical properties [24]. The tensile strength of silk fibroin is approximately 740 MPa, and its toughness is approximately 6 × 10^4^ J kg^−1^ [25]. Silk fibroin has been used to prepare good-biocompatibility and stretchable hydrogels for wearable flexible sensors [26,27]. For example, Paolieri et al. proposed a method to prepare hydrogel by coordination of oxidized silk fibroin, tannic acid, and polydopamine-modified Ti_3_C_2_ MXene nanosheets with ferric ions, which can be used as wearable pressure sensors [28]. On the other hand, various amino acid functional groups of silk fibroin can provide ion coordination and hydrogen bonding. However, current research on the dual network hydrogel constructed by ion crosslinking PAA and silk fibroin is still limited, yet such insight could provide potential value for wearable hydrogel sensors. In addition, hydrogels as a medium for the transmission of electrical signals need to have a good conductivity, which can be divided into ionic conductivity and electronic conductivity [29,30]. In order to improve the conductivity of hydrogels, some conductive media, such as graphene and carbon nanotubes, can be introduced into the hydrogel [31,32]. MXene, as a two-dimensional material, possesses excellent conductivity and energy storage capabilities, and has been used in the development of conductive hydrogels [33]. For example, Li et al. prepared a MXene-reinforced stretchable conductive hydrogels via a one-pot method [34]. Furthermore, the poor flexibility, low sensitivity, and instability of hydrogels limit their practical application in wearable sensing. Thus, the development of multifunctional hydrogels with high sensing performance for wearable flexible sensors is imperative, but also challenging.

Herein, we prepared a multifunctional hydrogel for wearable capacitive strain sensors through ion crosslinking. PAA is crosslinked by Zn^2+^ coordination to form a network structure, which, together with silk fibroin, constitutes hydrogel with a uniform dual network structure. Ti_3_C_2_T_x_ MXene, as a conductive medium, is introduced into the hydrogel, not only improving the conductivity of the hydrogel but also enhancing the stability of the dual network structure through hydrogen bonding. The prepared injectable hydrogel has good multifunctionality, such as high flexibility, rapid self-healing, and high conductivity. We further assembled the hydrogel into a capacitive strain sensor, which demonstrated high sensitivity and a wide working range in wearable sensing. When used for joint activity monitoring, the hydrogel showed the ability for precise differentiation of various signals, real-time responsiveness, and long-term operational stability. This method effectively improves the flexibility, self-healing, and injectability of multifunctional PAA-based hydrogels, which is expected to provide a new material for the field of wearable flexible sensors.

## 2. Results and Discussion

### 2.1. Preparation of PAA-Zn-Silk Fibroin-MXene Hydrogel

**Figure 1:**
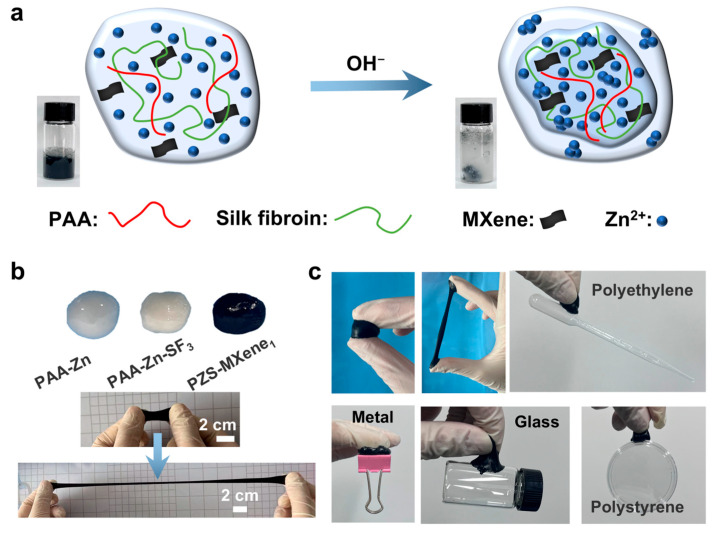
Schematic diagram and photos of the hydrogels. (**a**) Schematic diagram of the PZS-MXene hydrogel. The illustration showed crosslinking in the solution after adding sodium hydroxide. (**b**) Photographs and extensibility of hydrogels. (**c**) Adhesion of PZS-MXene hydrogel to different materials.

As shown in Figure 1a, the hydrogel is synthesized by the Zn^2+^ coordination. NaOH solution is added dropwise into the PAA solution, causing the PAA to deprotonate and generate a large amount of carboxylate, which can crosslink with Zn^2+^ through forming metal coordination bonds. Silk fibroin is added to the solution as a second network to stabilize the structure of the hydrogel. In addition, single layered Ti_3_C_2_T_x_ MXene nanosheets with high conductivity are introduced into the hydrogel as a conductive medium to improve conductivity. Crosslinked precipitates are collected in the reaction solution to obtain flexible and conductive PZS-MXene hydrogels. This method of using natural biological matrix to form hydrogel through ionic crosslinking is simple, effective, and more environmentally friendly than chemical crosslinking. We studied the hydrogels prepared with different amounts of silk fibroin and MXene, respectively. Photographs of hydrogels with different components are shown in Figure 1b. The prepared PZS-MXene hydrogel has ultra-high ductility and flexibility that can be folded, bent, and stretched (Figure S1a,c). In addition, the hydrogels can adhere well to different materials, such as metals, glass, and plastics (Figure 1c). When applied to fingers, the prepared PZS-MXene hydrogel can stick to the skin surface and stretch (Figure S1b). This is due to the abundant carboxyl groups of PAA-based hydrogel, which can form strong hydrogen bonding interactions with chemical groups on the interfaces of different materials, resulting in stable adhesion [35]. Furthermore, silk fibroin is rich in serine and tyrosine, which are also adhesives and can connect with substrates through hydrogen bonding [36]. Therefore, the prepared PZS-MXene hydrogel has good flexibility, stretchability, and adhesiveness, making it suitable for use in the field of wearable electronics.

### 2.2. Characterization of the Hydrogels

**Figure 2:**
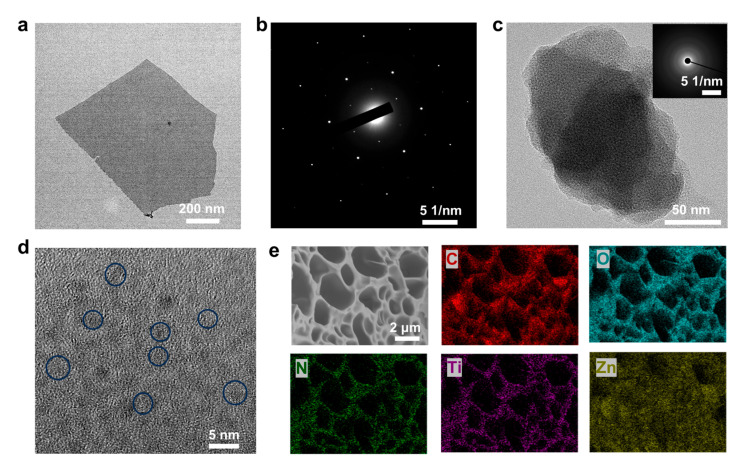
The microstructure of the PZS-MXene_1_ hydrogel. (**a**) TEM image of the Ti_3_C_2_T_x_ MXene nanosheet. (**b**) Corresponding electron diffraction of the Ti_3_C_2_T_x_ MXene nanosheet. (**c**) TEM image of the PZS-MXene_1_ hydrogel. The illustration shows the corresponding electron diffraction. (**d**) HRTEM image of the PZS-MXene_1_ hydrogel. (**e**) SEM image and elemental distribution of the PZS-MXene_1_ hydrogel.

As a two-dimensional nanomaterial, single-layer structured MXene is conducive to the uniform distribution of MXene in hydrogels and efficient charge transfer. We conducted transmission electron microscopy on the structure of Ti_3_C_2_T_x_ MXene. As shown in Figure 2a, the Ti_3_C_2_T_x_ MXene used has an obvious two-dimensional monolayer nanosheet structure, which is different from ordinary multi-layer MXene. The electron diffraction spots of the Ti_3_C_2_T_x_ MXene nanosheet show single crystal diffraction spots, indicating the hexagonal structure of Ti_3_C_2_T_x_ MXene on the atomic scale (Figure 2b). This nanosheet structure is conducive to the uniform dispersion of MXene in the hydrogel. The Ti_3_C_2_T_x_ MXene nanosheets can be used as an excellent conductive medium to improve the conductivity of hydrogels. At the same time, as a rigid material, Ti_3_C_2_T_x_ MXene nanosheets can play a role in strengthening and toughening hydrogels. The microstructure of the hydrogels was observed by fracturing in liquid nitrogen and freeze-drying. As shown in Figure S2, the Zn^2+^-crosslinked hydrogel has an interconnected porous network structure, which gives it good stretchability. It can be observed that with the increase in the concentration of silk fibroin, fibers are gradually formed in the pores of the hydrogel, forming a double network structure with the PAA chains. When the concentration of silk fibroin is 5%, the silk fibroin is not only dispersed inside the pore but also covers the surface of the pore. When the concentration of silk fibroin is 3%, the silk fibroin is evenly dispersed in the porous network structure. Then, the Ti_3_C_2_T_x_ MXene nanosheets were introduced into the hydrogel. As shown in Figure S3, at the MXene concentrations of 0.5% and 1%, the porous structure of the hydrogel is not destroyed, and there are no obvious agglomerates, indicating the uniform distribution of Ti_3_C_2_T_x_ MXene in the hydrogel. When the concentration of Ti_3_C_2_T_x_ MXene is further increased to 1.5%, agglomerates are formed in the hydrogel. The formation of such agglomerates hinders the continuity of the crosslinked networks, which is not conducive to the stretchability of the hydrogel. Therefore, when the concentration of silk fibroin is 3% and the concentration of Ti_3_C_2_T_x_ MXene is 1%, the prepared hydrogel has a uniform porous network structure. The PZS-MXene_1_ hydrogel, after freeze-drying, was further observed by transmission electron microscopy (Figure 2c). The corresponding electron diffraction diagram indicates that the amorphous structure of PZS-MXene_1_ hydrogel. As shown in Figure 2d, it can be observed that the PZS-MXene_1_ hydrogel contains nanoparticles with weak crystallization from the HRTEM image. These small nanoparticles are the reaction products of zinc ions and hydroxide ions, and are wrapped by crosslinked networks. As shown in Figure 2e, the PZS-MXene_1_ hydrogel has a porous network structure. Table S1 shows the mass percentage of each element within the hydrogel. In addition, the corresponding elemental mapping spectra show that the N, Ti, and Zn elements are evenly distributed in the hydrogel, indicating the uniform distribution of silk fibroin and Ti_3_C_2_T_x_ MXene in the network structure. These results indicate that the hydrogel with uniform porous structure is obtained.

**Figure 3:**
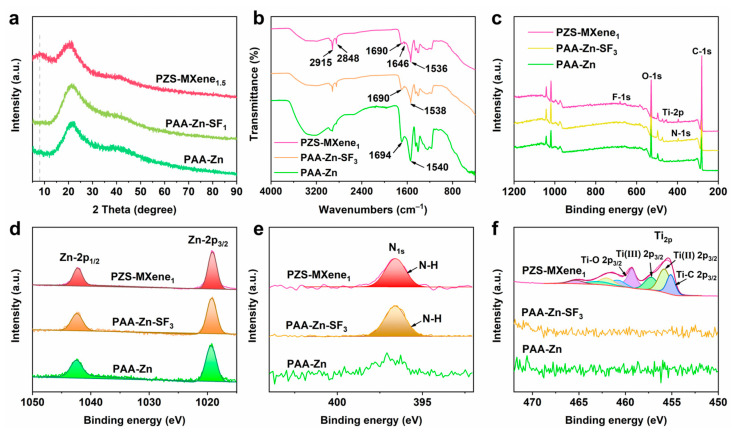
The characterization of the hydrogels. (**a**) XRD of the hydrogels. (**b**) FTIR of the hydrogels. (**c**) XPS spectrograms of the hydrogels. (**d**) High-resolution Zn-2p spectrum. (**e**) High-resolution N-1s spectrum. (**f**) High-resolution Ti-2p spectrum.

The XRD phase of prepared different hydrogels was conducted. As shown in Figure 3a and Figure S4, the prepared hydrogels are amorphous phases. In addition, compared to PAA-Zn and PAA-Zn-SF_1_, PZS-MXene_1.5_ hydrogel exhibits a peak envelope around 7°, which corresponds to the diffraction peak of Ti_3_C_2_T_x_ MXene. The results indicated that MXene is successfully introduced into the hydrogel. In addition, the chemical bonds in the hydrogels were further analyzed by Fourier transform infrared spectrometer. As shown in Figure 3b, the absorption peak at 2915 cm^−1^ corresponds to the asymmetric stretching vibration of the C-H bond of methylene, and the absorption peak at 2852 cm^−1^ corresponds to the symmetric stretching vibration of the C-H bond on methylene [37]. In PAA-Zn hydrogel, the absorption peak at 1694 cm^−1^ can be attributed to the stretching vibration of the carboxyl group (-COOH) [38]. After dropwise addition of NaOH and chelation with Zn^2+^, the carboxylate radical (-COO^-^) forms a vibration absorption peak at 1540 cm^−1^. The absorption peak of the carboxyl group shifts from 1694 cm^−1^ to 1690 cm^−1^, and the absorption peak of carboxylate shifts from 1540 cm^−1^ to 1538 cm^−1^ after adding silk fibroin, indicating the formation of hydrogen bonds between silk fibroin and PAA. This is due to the fact that silk fibroin is rich in various serine and tyrosine, which could connect with carboxyl groups through hydrogen bonds. After further adding Ti_3_C_2_T_x_ MXene, the absorption peak of carboxylate shifts to 1536 cm^−1^. This can be attributed to the hydroxyl groups on the surface of MXene nanosheets, which can form hydrogen bond interactions with the carboxylate groups of PAA chains. In addition, compared with PAA-Zn hydrogel, there is a new diffraction peak at 1646 cm^−1^ in PAA-Zn-SF_3_ and PZS-MXene_1_ hydrogels, which could be attributed to the amide group of silk fibroin. And the absorption peak of the amide group of PZS-MXene_1_ hydrogel is more obvious, indicating that there are hydrogen bond interactions between silk fibroin and MXene. The results show that the prepared PZS-MXene_1_ contains abundant hydrogen bond interactions, which is beneficial to improving the stability of the network structure.

The XPS was utilized to further analyze the binding energy of elements in the hydrogels. As shown in Figure 3c, the full spectrum indicates that the PZS-MXene hydrogel contains obvious N and Ti peaks, which are derived from silk fibroin and Ti_3_C_2_T_x_ MXene, respectively. As shown in Figure 3d, the peaks at 1042.3 eV and 1019.3 eV correspond to Zn-2p_1/2_ and Zn-2p_3/2_, respectively. Compared with PAA-Zn hydrogel, after adding silk fibroin, there is a distinct N-1s peak at 396.6 eV for PAA-Zn-SF_3_ and PZS-MXene_1_ hydrogels (Figure 3e). When Ti_3_C_2_T_x_ MXene is further added, PZS-MXene_1_ hydrogel has an obvious Ti-2p peak (Figure 3f). The results show that Ti_3_C_2_T_x_ MXene is stably introduced into the hydrogel, which is consistent with the XRD results.

### 2.3. Stretchability and Rheological Properties of the Hydrogels

**Figure 4:**
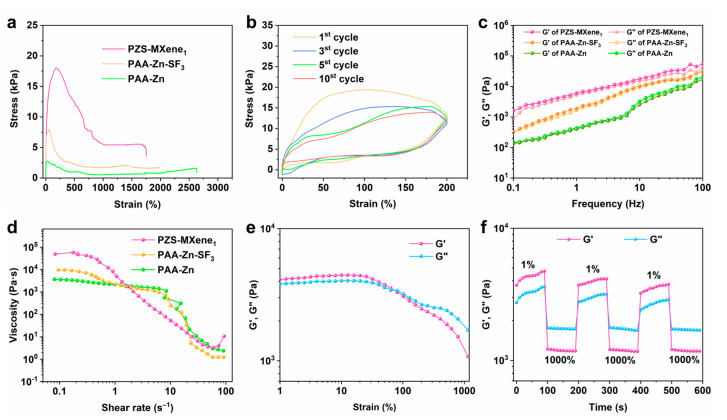
Stretchability and rheological properties of hydrogels. (**a**) Tensile fracture stress–strain curves of hydrogels. (**b**) Cyclic tensile stress–strain curves of PZS-MXene_1_ hydrogel under 200% strain. (**c**) Storage modulus (G’) and loss modulus (G”) of hydrogels at different frequencies. (**d**) Viscosity changes of hydrogels at different shear rates. (**e**) G’ and G” of hydrogels at different strain ranges. (**f**) G’ and G” of hydrogels at different strain cycles.

Hydrogel can be used as strain sensor due to its excellent ductility. Good stretchability can meet the requirements of maintaining the stable structure of the hydrogel under deformation. As shown in Figure 4a, the tensile properties of the prepared PAA-Zn, PAA-Zn-SF_3_, and PZS-MXene_1_ hydrogels were tested. The Zn^2+^–crosslinked PAA-Zn hydrogel has a high strain of 2630%, but its tensile stress is 2.7 kPa, showing the good flexibility but poor stiffness of the hydrogel. This is due to the poor crosslinking density of the PAA-Zn hydrogel, which is not conducive to structural recovery. After adding silk fibroin, the fracture strain of PAA-Zn-SF_3_ hydrogel is 1986%. As a result of entanglement of silk fibroin and PAA chains, the fracture stress increases to 7.9 kPa. After further introducing the Ti_3_C_2_T_x_ MXene nanosheet into the hydrogel, the fracture strain of PZS-MXene_1_ hydrogel is 1750%, and the fracture stress is 18.1 kPa. The results indicate that Ti_3_C_2_T_x_ MXene nanosheet can further improve the tensile properties of the hydrogel. This is due to the fact that Ti_3_C_2_T_x_ MXene, as a rigid material, is filled into the hydrogel, thereby increasing the crosslinking density of the networks and strengthening the stiffness of the hydrogel. From the tensile stress–strain curve, it can be observed that the elastic deformation of the hydrogel occurs within 200% strain, and the stress reaches 18.1 kPa. The internal structure of the PZS-MXene_1_ hydrogel was gradually destroyed, and plastic deformation was produced during the stretching process, which made the stress decrease. This is due to the presence of a large number of metal coordination bonds and hydrogen bonds in the hydrogel; these dynamic bonds can play the role of energy dissipation, thereby improving the stretchability of the hydrogel [39,40]. On the other hand, the dual structure of the silk fibroin and PAA network in the hydrogel dissipates energy and increases the energy required for the hydrogel to completely break [41]. The cyclic tensile property of the PZS-MXene_1_ hydrogel was further tested. As shown in Figure 4b, at 200% cyclic strain, the tensile and recovery curves of the hydrogel do not overlap, showing the energy dissipation capacity of the hydrogel. This can be attributed to the large amount of hydrogen bonds and metal coordination bonds in the PZS-MXene_1_ hydrogel, which can absorb energy through the breaking of hydrogen bonds and ion bonds during the stretching process to improve the toughness of the hydrogel. In addition, at the 5th and 10th cycles, the stress–strain curves almost overlap, indicating the tensile cycle stability of the PZS-MXene_1_ hydrogel.

The rheological properties of the hydrogels were further characterized by a rheometer. The storage modulus (G’) and loss modulus (G”) of hydrogels were tested from 0.1 to 100 Hz frequency range at a strain of 0.2%. As shown in Figure 4c, the result shows that all the hydrogels are liquid-like since the moduli are highly frequency-dependent. After adding silk fibroin, the G’ and G” of PAA-Zn-SF_3_ hydrogel is higher than that of PAA-Zn, which can be due to the formation of double network structure. Besides, the G’ of PAA-Zn hydrogel is slightly lower than the G”, indicating the weak crosslinking of hydrogels. In comparison, Ti_3_C_2_T_x_ MXene makes the double network structure of PZS-MXene_1_ hydrogel more stable, resulting in the obvious improvement in G’ and G”. Moreover, the G’ of PZS-MXene_1_ hydrogel is higher than the G”. The results show that the PZS-MXene_1_ hydrogel is strongly crosslinked. Therefore, it can be concluded that silk fibroin can form a more complex network structure. Meanwhile, MXene improves the stability of network structure. The relationship between the viscosity and the shear rate of hydrogels was further studied. As shown in Figure 4d, with the increase in shear rate, the viscosity decreases gradually, showing that the hydrogel has shear thinning characteristics. This shear thinning ability of hydrogel enables it to have good plasticity, so it is easy to be processed into any complex shape.

To further analyze the self-healing properties of PZS-MXene_1_ hydrogels, the G’ and G” were tested from 1% to 1000% strain at a frequency of 1 Hz (Figure 4e). Within the 1–100% strain range, the G’ is higher than the G”, indicating the solid-like behavior of the PZS-MXene_1_ hydrogel. After the shear strain is higher than 100% strain, the G’ is lower than the G”, indicating the liquid-like behavior due to the destruction of structure under dynamic testing. Then, the self-healing properties of the PZS-MXene_1_ hydrogel were tested by dynamic frequency sweep under cyclic strain. As shown in Figure 4f, at 1% strain, the G’ is higher than the G”. At 1000% strain, the G’ is lower than the G”. It can be observed that when it returns to 1% strain, the G’ and G” quickly return to their initial solid-like state. After three cycles, the G’ and G” have no obvious changes, showing the good self-healing performance of the PZS-MXene_1_ hydrogel. This is due to a large number of dynamic ionic coordination bonds formed by Zn^2+^ and PAA in the hydrogel, which allows the structure to self-heal quickly after damage. In addition, the hydrogen bonding formed between silk and Ti_3_C_2_T_x_ MXene in the hydrogel also contributes to improving the self-healing properties of the hydrogel.

### 2.4. Self-Healing Ability and Conductivity of the Hydrogels

**Figure 5:**
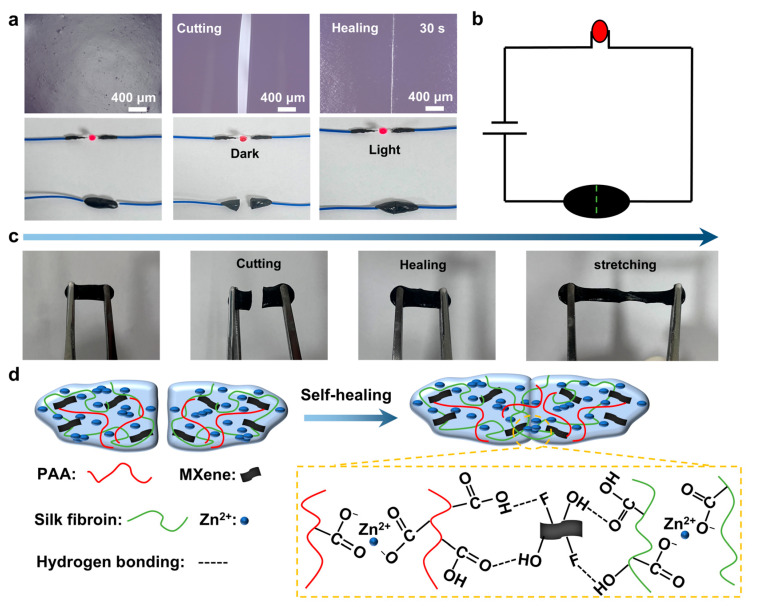
Self-healing test of the PZS-MXene_1_ hydrogel. (**a**) Self-healing process with an ultra-depth 3D microscope and a diode indicator light. (**b**) Schematic diagram of hydrogel pathway. (**c**) Flexibility of hydrogels after self-healing. (**d**) Schematic diagram of self-healing mechanism of PZS-MXene_1_ hydrogel.

As shown in Figure 5a, the self-healing behavior of the PZS-MXene_1_ hydrogel was observed under an ultra-depth 3D microscope. The initial hydrogel was cut and then re-pasted together. The hydrogel was tightly bonded together after 30 s, and the crack almost completely disappeared. The results show that the PZS-MXene_1_ hydrogel has a rapid self-healing property. Moreover, the hydrogel is conductive and can light up a diode indicator when connected to a power source (Figure 5b). When the hydrogel is cut, the circuit is broken, and the diode indicator goes out. When the hydrogel is pasted back together, the diode indicator lights up again. It can be concluded that the PZS-MXene_1_ hydrogel maintains good conductivity after self-healing. As shown in Figure 5c, the PZS-MXene_1_ hydrogel can still be stretched after cutting and re bonding. The tensile property of PZS-MXene_1_ hydrogel after self-healing was measured (Figure S5). PZS-MXene_1_ hydrogel was cut off and re-bonded together for 5 min before being used for the tensile fracture test. The test results show that the PZS-MXene_1_ hydrogel can still maintain a high tensile strength of 1200% after self-healing, and the tensile stress is 12.9 kPa. After self-healing, the hydrogel still has ductility and flexibility, which corresponds to the rheological test results. The self-healing mechanism of PZS-MXene_1_ hydrogel is illustrated in Figure 5d. Among them, the PZS-MXene_1_ hydrogel contains a large number of ion coordination bonds formed by Zn^2+^ and carboxyl (-COO^−^) groups of PAA chains. In addition, the hydroxyl (-OH) and fluorine (-F) functional groups on the surface of Ti_3_C_2_T_x_ MXene, as well as the hydroxyl (-OH) and carboxyl (-COO^−^) groups of silk fibroin fibers, can form hydrogen bonds. These ionic coordination bonds and hydrogen bonds enable hydrogels to have instant self-healing ability.

**Figure 6:**
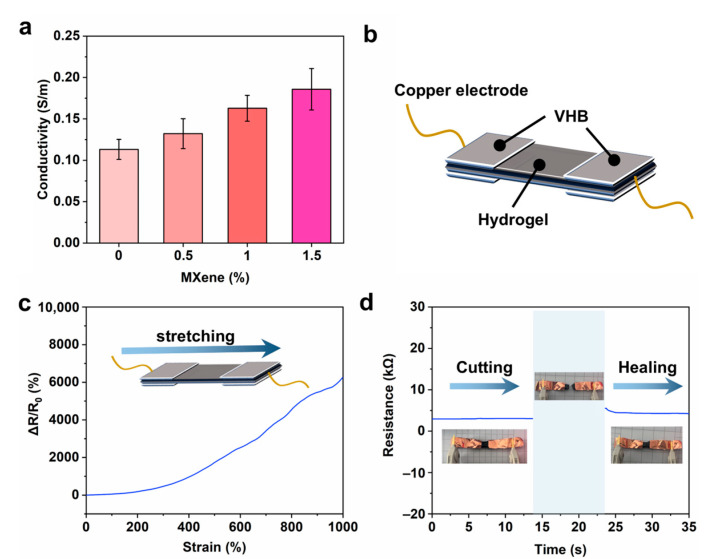
(**a**) Conductivity of the hydrogels. (**b**) Schematic diagram of resistance and strain response test. (**c**) Corresponding resistance and strain response curve of PZS-MXene_1_ hydrogel. (**d**) Resistance changes after self-healing.

MXene, as an emerging two-dimensional energy storage material, has excellent conductivity and energy storage, and can be used to prepare flexible capacitors [42]. Therefore, the conductivity of hydrogels with different Ti_3_C_2_T_x_ MXene concentrations was tested. As shown in Figure 6a, the preparation involves the use of ZnCl_2_ and NaOH, which leads to a high content of ions such as sodium ions and chloride ions in the hydrogel. These ions can endow the crosslinked polymer matrix with significant ionic conductivity [43,44]. Therefore, the initial conductivity of the hydrogel can be measured as 0.11 S/m. With the increase in Ti_3_C_2_T_x_ MXene concentration, the conductivity of the PZS-MXene hydrogel gradually increases. This can be attributed to the introduction of Ti_3_C_2_T_x_ MXene with high electronic conductivity into the hydrogel, which can synergistically improve the conductivity of the hydrogel [45]. It can be known from the characterization that when the Ti_3_C_2_T_x_ MXene concentration is 1%, the structure of the hydrogel is the most uniform, and the conductivity is increased to 0.16 S/m. As illustrated in Figure 6b, the two ends of the PZS-MXene_1_ hydrogel are connected to copper electrodes through VHB to further test the resistance and strain response. Due to the good conductivity of PZS-MXene_1_ hydrogel, stable resistance changes can occur under different strains (Figure 6c). When the strain increases from 0 to 1000%, the relative resistance of the PZS-MXene_1_ hydrogel increases significantly from 0 to 6200%. The results show that the resistance of the hydrogel depends on the strain, which can be attributed to the high extensibility and conductivity of the prepared PZS-MXene_1_ hydrogel. The quantitative changes in the resistance of the PZS-MXene_1_ hydrogel were further tested by an LCR meter (Figure 6d). It can be observed that the initial resistance of the PZS-MXene_1_ hydrogel is about 3 kΩ. In addition, the resistance quickly returns to around 3 kΩ after cutting and re-contacting, showing the good self-healing ability of the PZS-MXene_1_ hydrogel.

### 2.5. Capacitive Strain Sensing Performance of the Hydrogels

**Figure 7:**
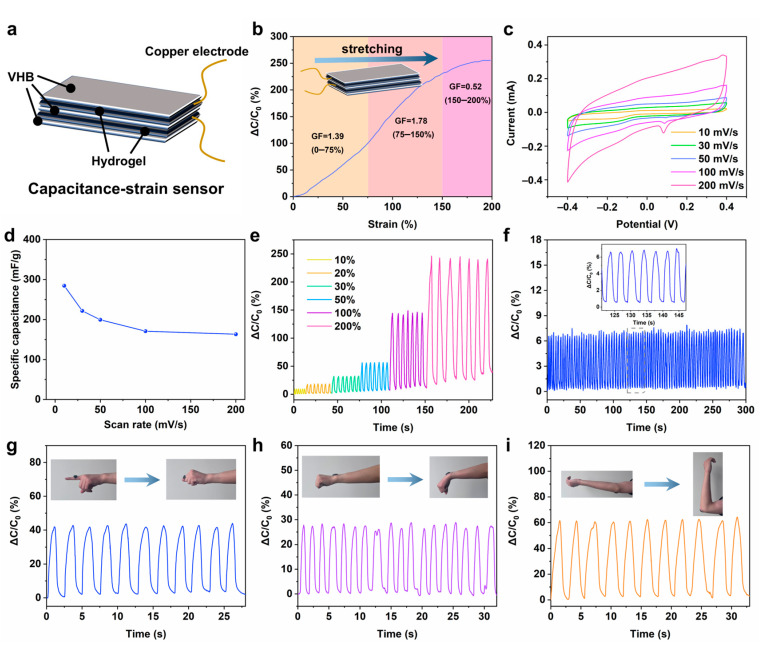
(**a**) Assembly diagram of capacitive strain sensor. (**b**) Sensitivity of the capacitive strain sensing. (**c**) Cyclic voltammetry curves at different scanning speeds. (**d**) Capacitance of hydrogel at different scanning rates. (**e**) Relative capacitance response under different cyclic strains. (**f**) Relative capacitance response under long cycles. (**g**) Finger motion monitoring. (**h**) Wrist movement monitoring. (**i**) Elbow bending monitoring.

At present, most of the hydrogel sensors are of the resistance response type. In order to realize the application of hydrogel in multifunctional sensors, it is of great significance to develop capacitive sensors. In this work, we assembled the prepared PZS-MXene_1_ hydrogel into a flexible capacitive strain sensor. As shown in Figure 7a, a layer of VHB is sandwiched between two pieces of hydrogel, and the side of the VHB with copper foil is attached to the surface of the upper and lower hydrogels to package into a capacitive strain sensor. This VHB as a flexible tape can well encapsulate the hydrogel and prevent the volatilization of water. As shown in Figure 7b, the capacitive sensing performance of hydrogel was tested. When the tensile strain range increased from 0% to 200%, the relative capacitance of the hydrogel increased from 0% to 250%, indicating that the hydrogel has a good capacitive strain response. The gauge factor was calculated by calculating the slope of the capacitance strain curve to assess the sensitivity of the PZS-MXene_1_ hydrogel. It can be obtained that in the smaller strain range of 0–75%, the gauge factor is 1.39. In the strain range of 75–150%, the gauge factor is 1.78. In the higher strain range of 150–200%, the gauge factor is 0.52. The results show that the prepared PZS-MXene_1_ hydrogel has a high sensitivity. In addition, fast response is an important indicator of sensor performance. The response time of the PZS-MXene_1_ hydrogel capacitive strain sensor was measured at 50% strain (Figure S6). The response time of the capacitive strain sensor is 0.2 s, and the relaxation time is 0.35 s, indicating the fast responsiveness of PZS-MXene_1_ hydrogel, which can ignore the signal lag. As shown in Table 1, we compared the key performance indicators of other hydrogel capacitive strain sensors with this work. The prepared capacitive strain sensors have poor conductivity, low sensitivity, and low working strains, which limits their practical application in wearable sensing [11,38,39]. By comparison, it can be concluded that the sensing performance of PZS-MXene_1_ hydrogel prepared in this work is higher than most of the state-of-the-art hydrogels, showing high sensitivity, wide working strain, and fast responsiveness. On the other hand, the preparation process of most reported hydrogels is complex and harsh, such as the use of a large number of initiators or crosslinking agents in chemical crosslinking, which may lead to a certain degree of biosafety hazards of this kind of hydrogels, and also affect the dynamic adjustability of the crosslinked structure [40,41,42]. In this work, natural silk fibroin was used to prepare the hydrogel by ionic crosslinking, which is simple and biocompatible. In addition, the structure and properties of ion-crosslinked hydrogels are dynamically tunable.

The cyclic voltammetry of the PZS-MXene_1_ hydrogel was measured at different sweep rates from −0.4 V to 0.4 V. As shown in Figure 7c, all the curves keep a similar rectangular shape with increasing scan rate, indicating the good capacitive character of PZS-MXene_1_ hydrogel. The reduction peak at 0.1 V can be attributed to the reduction of Zn^2+^ in the hydrogel. The capacitance of PZS-MXene_1_ hydrogel at different scanning speeds was calculated (Figure 7d). When the scanning rate is 10 mV/s, the capacitance of the PZS-MXene_1_ hydrogel is 284.6 mF/g. When the scanning speed increases to 200 mV/s, the capacitor still has 163.2 mF/g. The results show that the PZS-MXene_1_ hydrogel exhibits the energy storage capability. Furthermore, the capacitive sensing performance was further tested under different cyclic strains. As shown in Figure 7e, with the cyclic strain gradually increasing from 10% to 200%, the relative capacitance of PZS-MXene_1_ hydrogel significantly increases from 7% to 250%. Benefitting from the good sensitivity, the PZS-MXene_1_ hydrogel shows a good accurate recognition ability for different strains. The long-term work stability was tested (Figure 7f). It can be found that the PZS-MXene_1_ hydrogel maintains a stable relative capacitance change within 100 times of continuous fast cyclic stretching, showing good stability, which can be used for the monitoring of repeated joint activities. As shown in Figure S7, after a long period of sensing, the strain sensor maintains its intact structure. By observing the microstructure, it can be found that the PZS-MXene_1_ hydrogel still has a uniform porous structure after use. This is due to the reduced water loss after VHB encapsulation and the self-healing ability of the hydrogel, which makes the structure of the hydrogel remain stable. Furthermore, natural silk fibroin can provide a stable crosslinking network and guarantee the transmission of charge in the hydrogel, which is conducive to the stable conductivity of the hydrogel [27,51]. On the other hand, the residues of silk fibroin can also form ion interactions to improve the electrical properties of hydrogels [52,53]. The PZS-MXene_1_ hydrogel is applied to different joint parts to test its real-time monitoring performance of joint activities. As shown in Figure 7g, when attached to the finger joint, a stable relative capacitance change can be generated following the bending of the finger. In addition, the hydrogel is pasted on the wrist (Figure 7h). With the wrist bending down, the hydrogel produces a real-time capacitive signal change. As shown in Figure 7i, the hydrogel is adhered to the elbow. With the elbow bending up, the relative capacitance of the hydrogel increases from 0% to 60%. When it returns to its initial state, the relative capacitance returns to 0%. These results show that the PZS-MXene_1_ hydrogel capacitive strain sensor has the ability to respond to different joint activities in real-time, showing good stability and accuracy. These performances make the hydrogel promising to be used in the field of wearable flexible electronics.

## 3. Conclusions

In summary, we prepared a flexible PZS-MXene_1_ hydrogel by ionic crosslinking. PAA and Zn^2+^ form a crosslinked network through ion coordination, and further form a double network structure with silk fibroin. After adding Ti_3_C_2_T_x_ MXene, the prepared injectable PZS-MXene_1_ hydrogel has a uniform porous network structure, good flexibility, (1750% elongation) and a fast self-healing property (30 s). The PZS-MXene_1_ hydrogel exhibits an enhanced conductivity (0.16 S/m). The capacitive strain sensor assembled with PZS-MXene_1_ hydrogel shows high sensitivity (GF = 1.78) and wide operating strain range (0–200%). Under tensile deformation, the PZS-MXene_1_ hydrogel sensor has accurate response and good cycle stability. As a wearable flexible device, it can monitor the movement of different joint activities in real time, such as fingers, wrists, and elbows. Our approach is expected to be applied in the field of wearable electronics.

## 4. Materials and Methods

### 4.1. Materials

Polyacrylic acid (PAA, average Mw ~250,000, 35 wt. % in H_2_O), was purchased from Sigma-Aldrich (St. Louis, MO, USA). Zinc chloride (ZnCl_2_, AR), sodium hydroxide (NaOH, AR), and lithium bromide (LiBr, AR) were obtained from Sinopharm Chemical Reagent Co., Ltd. (Shanghai, China). Single layered Ti_3_C_2_T_x_ MXene dispersion (10 mg/mL) were purchased from FoShan Xinxi Technology Co., Ltd. (FoShan, Guangdong, China). Degummed silk was obtained from Simatech Incorporation (Suzhou, China).

### 4.2. Preparation of Silk Fibroin Solution

First, 80.77 g of LiBr was dissolved in 60 mL deionized water, and then diluted to a volume of 100 mL to obtain a 9.3 M LiBr solution. The LiBr solution was left to stand for 3 days at room temperature. A quantity of 100 mL LiBr solution was heated to 60 °C, and 25 g of degummed silk was added and slowly stirred for 2 h. The silk fibroin solution was placed in a 14 kDa dialysis bag and dialyzed in 1 L deionized water for 3 days, with the deionized water changed every 8 h. The silk fibroin solution obtained after dialysis was centrifuged at 9000 r/min for 15 min to remove the precipitate. The mass concentration of silk fibroin was calculated by drying, and the silk fibroin was stored at 4 °C.

### 4.3. Preparation of PAA-Zn-SF-MXene Hydrogels

First, the 0.1 M NaOH solution was prepared for use, and a 0.1 M ZnCl_2_ and 0.2 M PAA mix solution was prepared. The silk fibroin solution with 3% mass concentration was added to the 25 mL ZnCl_2_ and PAA mix solution under stirring, and it was mixed for 20 min. Ti_3_C_2_T_x_ MXene nanosheet solution was sonicated for 5 min in an ice bath, and then Ti_3_C_2_T_x_ MXene with 1% mass concentration was added to the mix solution and stirred for 20 min under ice bath. A quantity of 25 mL NaOH solution was slowly added to the mix solution while slowly stirring. Then, the solution was continuously slowly stirred for 1 h to form a black hydrogel. The residual solution was discarded, and the PAA-Zn-SF-MXene hydrogel was repeatedly cleaned with deionized water several times. Pure PAA-Zn hydrogel was also prepared in the same way. The hydrogels prepared by adding silk fibroin with different mass concentrations of 1%, 3%, and 5% are named as PAA-Zn-SF_1_, PAA-Zn-SF_3_, and PAA-Zn-SF_5_, respectively. The hydrogels prepared by adding Ti_3_C_2_T_x_ MXene with different mass concentrations of 0.5%, 1%, and 1.5% are named as PZS-MXene_0.5_, PZS-MXene_1_, and PZS-MXene_1.5_, respectively.

### 4.4. Characterization of Hydrogels

The hydrogel was quenched in liquid nitrogen and freeze-dried for characterization and analysis. The micromorphology of the hydrogel was observed by scanning electron microscope (SEM, SU8230, Hitachi, Ltd., Tokyo, Japan) at 5 kV and 10 μA. Freeze-dried hydrogels were ground and crushed, and then ultrasonically dispersed in alcohol solution. The internal microstructure of the sample was observed under a transmission electron microscope (TEM, JEM-F200, JEOL Ltd., Tokyo, Japan) at 120 kV. The phase of the hydrogel was analyzed by X-ray diffractometer (XRD, PANalytical-Empyrean X-ray diffractometer equipped, Malvern Panalytical, Malvern, UK) in the range of 5° to 90°. The chemical functional groups of the hydrogel were tested by Fourier transform infrared spectroscopy (FTIR, Thermo Nicolet Nexus, Ontario, ON, Canada) in the range of 4000 cm^−1^ to 400 cm^−1^. The element binding energy of hydrogels was obtained by X-ray photoelectron spectroscopy (XPS, ESCALAB 250Xi Thermo Fisher Scientific, Waltham, MA, USA).

### 4.5. Stretchability, Rheological Properties, and Self-Healing Ability Test of Hydrogels

The prepared hydrogel samples were cut into shapes of 5 cm in length, 1 cm in width and 3 mm in thickness. Then, the hydrogel was clamped in the universal mechanical testing machine (Instron 5967, Boston, MA, USA). The tensile properties of the hydrogel were tested at the deformation rate of 100 mm/min under ambient temperature. The cyclic tensile property of the PZS-MXene_1_ hydrogel was tested at the deformation rate of 50 mm/min under 200% strain.

The hydrogel was cut into a shape with a diameter of 20 mm and a thickness of 2 mm. The rheological properties of hydrogels were tested by rheometer. The storage modulus (G’) and loss modulus (G”) of hydrogels were measured from 0.1–100 Hz under 0.2% strain. The viscosity changes of hydrogels were measured from 0.1–100 s^−1^ under 0.2% strain. At a fixed frequency of 1 Hz, the viscoelastic response of the hydrogel was measured from 1% to 1000% of the strain range. For the self-healing test, the modulus change of the hydrogel was tested cyclically at 1% and 1000% strain. The interval between two successive cycles is 100 s. The self-healing process of PZS-MXene_1_ hydrogel was observed after cutting under an ultra-depth 3D microscope (Keyence, VHX-5000, Osaka, Japan).

### 4.6. Conductivity Test of Hydrogels

The hydrogel was cut into 2 cm long, 2 cm wide, and 5 mm thick shapes. The hydrogel was placed between two copper foil electrodes, and the resistance of the hydrogel was tested by LCR instrument (TH2830, Changzhou Tonghui Electronic Co. Ltd., Changzhou, China). The conductivity (σ)of the hydrogel is calculated as

σ = L/RS

where R is the measured resistance, L is the thickness of hydrogel, and S represents the contact area of hydrogel with copper foil.

### 4.7. Capacitive Strain Sensing Performance Test of Hydrogels

First, the PZS-MXene_1_ hydrogel was assembled into a capacitive strain sensor. Two identical hydrogels were sandwiched with VHB. Then, the VHB with copper electrode was pasted on the hydrogel and packaged as a capacitive strain sensor. The capacitance signal was received through a LCR instrument (TH2830, Changzhou Tonghui Electronic Co. Ltd., Changzhou, China).

### 4.8. Cyclic Voltammetry Test of Hydrogels

The cyclic voltammetry was measured by a CHI760E electrochemical workstation (Shanghai Chenhua Co., Ltd., Shanghai, China). The PZS-MXene_1_ hydrogel with a diameter of 10 mm was used as the working electrode. The reference electrode was Hg/HgO, the counter electrode was graphite, and the electrolyte was 1 M NaOH solution.

## Figures and Tables

**Table 1:** Comparison of sensing performance between this work and other hydrogel capacitive strain sensors.

Samples	Strain	Gauge Factor	Response Time	Conductivity	References
Starch/Poly(vinyl alcohol)/Borax/Carbon Nanotube	100%	0.91	0.2 s	0.08 mS/m	[11]
Poly(MAAc-*co*-NPAM)/NaCl	100%	0.19	0.4 s	2.51 S/m	[46]
PAA-r-BVIT/PEO	300%	1.1	0.08 s	0.036 S/m	[47]
NaSS/DMC Polyampholyte	350%	2.9	0.25 s	1.5 S/m	[48]
Sodium hyaluronate/polyacrylamide/glycerin/LiCl	190%	0.49	-	5 S/m	[49]
Zn-alginate/PAM	100%	0.8	-	3.24 S/m	[50]
PAA-Zn-SF-MXene	200%	1.78	0.2 s	0.16 S/m	This work

## Data Availability

The data presented in this study are openly available in the article.

## Supplementary Materials

The following supporting information can be downloaded at: https://www.mdpi.com/article/10.3390/gels11050377/s1, Figure S1. (a) Flexibility of hydrogel under bending and rotation. (b) Adhesion of hydrogel to skin. (c) Extension of the hydrogel. Table S1. Mass percentage of each element within the hydrogel. Figure S2. The SEM images of the hydrogels with different concentrations of silk fibroin (1%, 3% and 5%). Figure S3. The SEM images of the hydrogels with different concentrations of MXene (0.5%, 1% and 1.5%). Figure S4. XRD of the hydrogels. Figure S5. Tensile property of PZS-MXene_1_ hydrogel after self-healing. Figure S6. Response time of the PZS-MXene_1_ hydrogel capacitive strain sensor. Figure S7. (a) Hydrogel capacitive strain sensor before use (top) and after use (bottom). (b) The SEM image of the hydrogel after use.
