# Supplementary material for: Bio-Inspired Synthesis of Injectable, Self-Healing PAA-Zn-Silk Fibroin-MXene Hydrogel for Multifunctional Wearable Capacitive Strain Sensor
Source: Gels. 2025 May 21;11(5):377. doi: 10.3390/gels11050377 (PMC12111058; doi:10.3390/gels11050377)
Supplement: Supplementary file 1 [file gels-11-00377-s001.zip › gels-3652590-supplementary.pdf]

# Supporting Information

## Bio-inspired synthesis of Injectable, Self-healing PAA-Zn-Silk Fibroin-MXene Hydrogel for Multifunctional Wearable Capacitive Strain Sensor

**Rongjie Wang, Boming Jin, Jiaxin Li, Jing Li, Jingjing Xie, Pengchao Zhang, Zhengyi Fu**

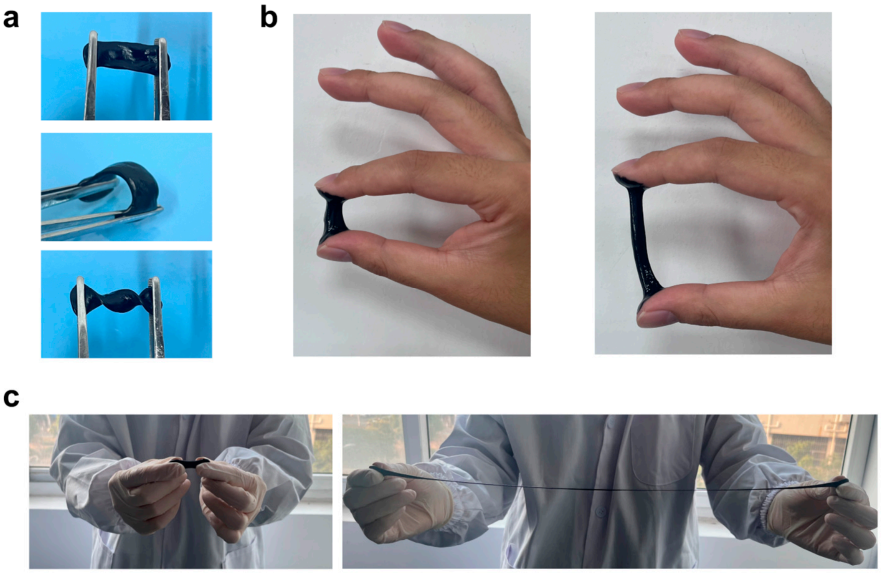


**Figure S1.** (a) Flexibility of hydrogel under bending and rotation. (b) Adhesion of hydrogel to skin. (c) Extension of the hydrogel.

**Table S1.** Mass percentage of each element within the hydrogel.

| Element | Wt% |
|---|---|
| C | 23.2 |
| O | 36.11 |
| N | 2.17 |
| Ti | 3.71 |
| Zn | 34.81 |

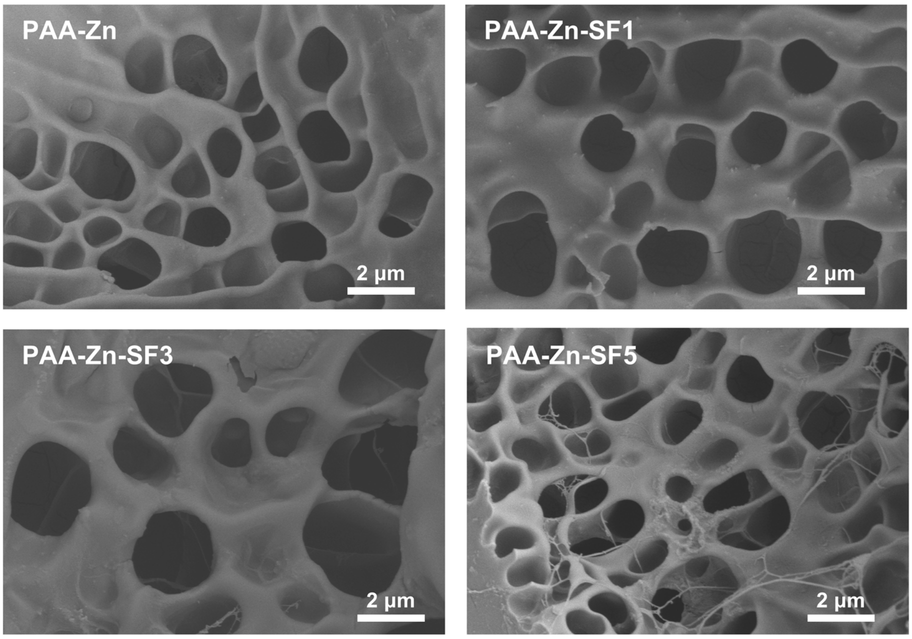


**Figure S2.** The SEM images of the hydrogels with different concentrations of silk fibroin (1%, 3% and 5%).

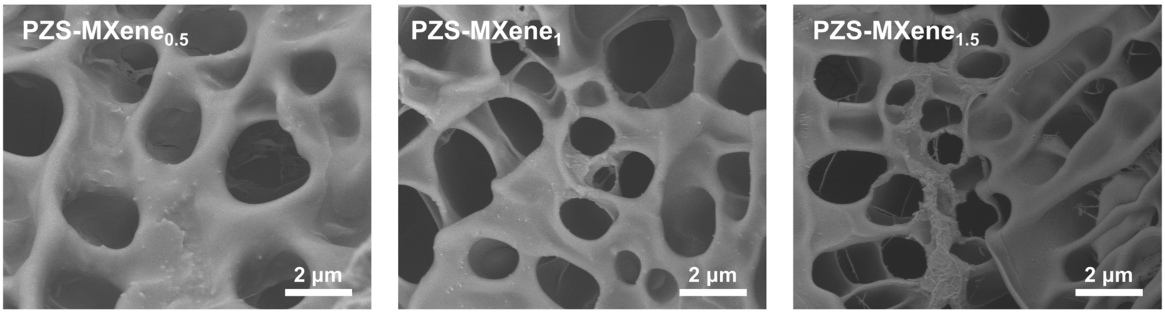


**Figure S3.** The SEM images of the hydrogels with different concentrations of MXene (0.5%, 1% and 1.5%).

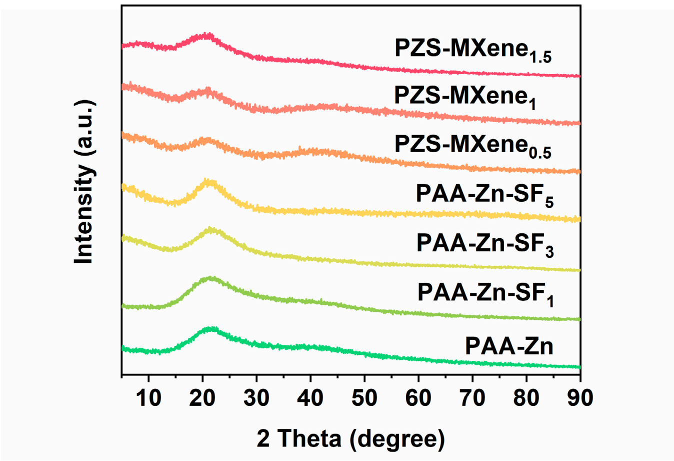


**Figure S4.** XRD of the hydrogels.

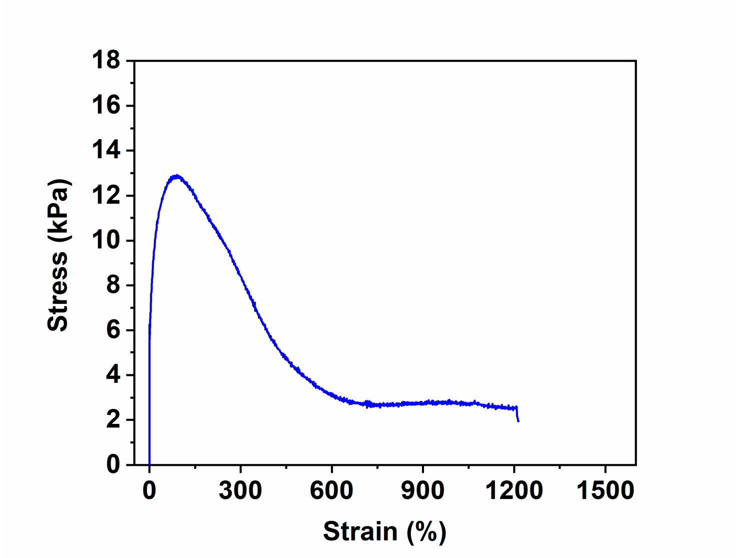

**Figure S5.** Tensile property of PZS-$MXene_1$ hydrogel after self-healing.

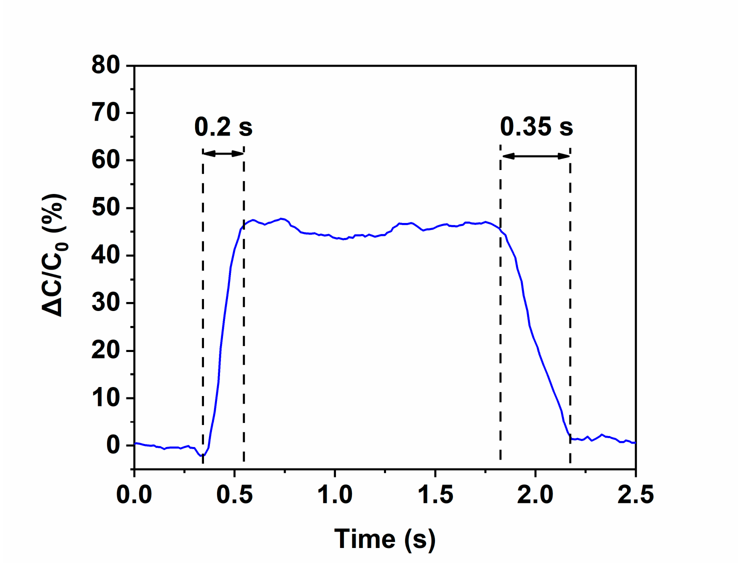


**Figure S6.** Response time of the PZS-$MXene_1$ hydrogel capacitive strain sensor.

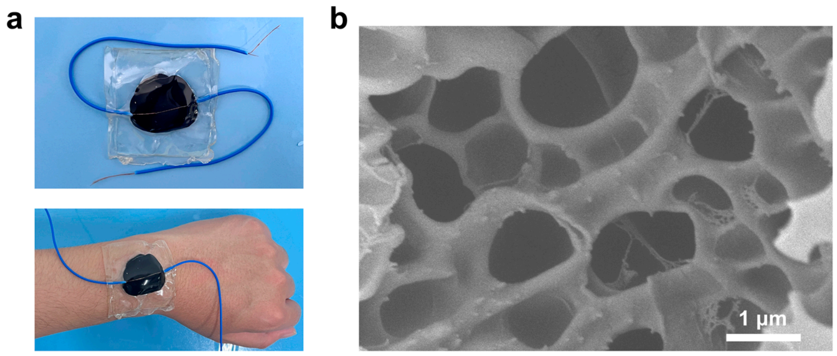


**Figure S7.** (a) Hydrogel capacitive strain sensor before use (top) and after use (bottom). (b) The SEM image of the hydrogel after use.
